# Mitochondria as a target and central hub of energy division during cold stress in insects

**DOI:** 10.1186/s12983-021-00448-3

**Published:** 2022-01-06

**Authors:** Jan Lubawy, Szymon Chowański, Zbigniew Adamski, Małgorzata Słocińska

**Affiliations:** 1 Department of Animal Physiology and Developmental Biology, Institute of Experimental Biology, Faculty of Biology, Adam Mickiewicz University, Poznan, Poland; 2 Laboratory of Electron and Confocal Microscopy, Faculty of Biology, Adam Mickiewicz University, Poznan, Poland

**Keywords:** Mitochondria, Cold stress, Bioenergetics, Enzymes activity, ATP, UCP, HSP, Apoptosis

## Abstract

Temperature stress is one of the crucial factors determining geographical distribution of insect species. Most of them are active in moderate temperatures, however some are capable of surviving in extremely high as well as low temperatures, including freezing. The tolerance of cold stress is a result of various adaptation strategies, among others the mitochondria are an important player. They supply cells with the most prominent energy carrier—ATP, needed for their life processes, but also take part in many other processes like growth, aging, protection against stress injuries or cell death. Under cold stress, the mitochondria activity changes in various manner, partially to minimize the damages caused by the cold stress, partially because of the decline in mitochondrial homeostasis by chill injuries. In the response to low temperature, modifications in mitochondrial gene expression, mtDNA amount or phosphorylation efficiency can be observed. So far study also showed an increase or decrease in mitochondria number, their shape and mitochondrial membrane permeability. Some of the changes are a trigger for apoptosis induced via mitochondrial pathway, that protects the whole organism against chill injuries occurring on the cellular level. In many cases, the observed modifications are not unequivocal and depend strongly on many factors including cold acclimation, duration and severity of cold stress or environmental conditions. In the presented article, we summarize the current knowledge about insect response to cold stress focusing on the role of mitochondria in that process considering differences in results obtained in different experimental conditions, as well as depending on insect species. These differentiated observations clearly indicate that it is still much to explore.

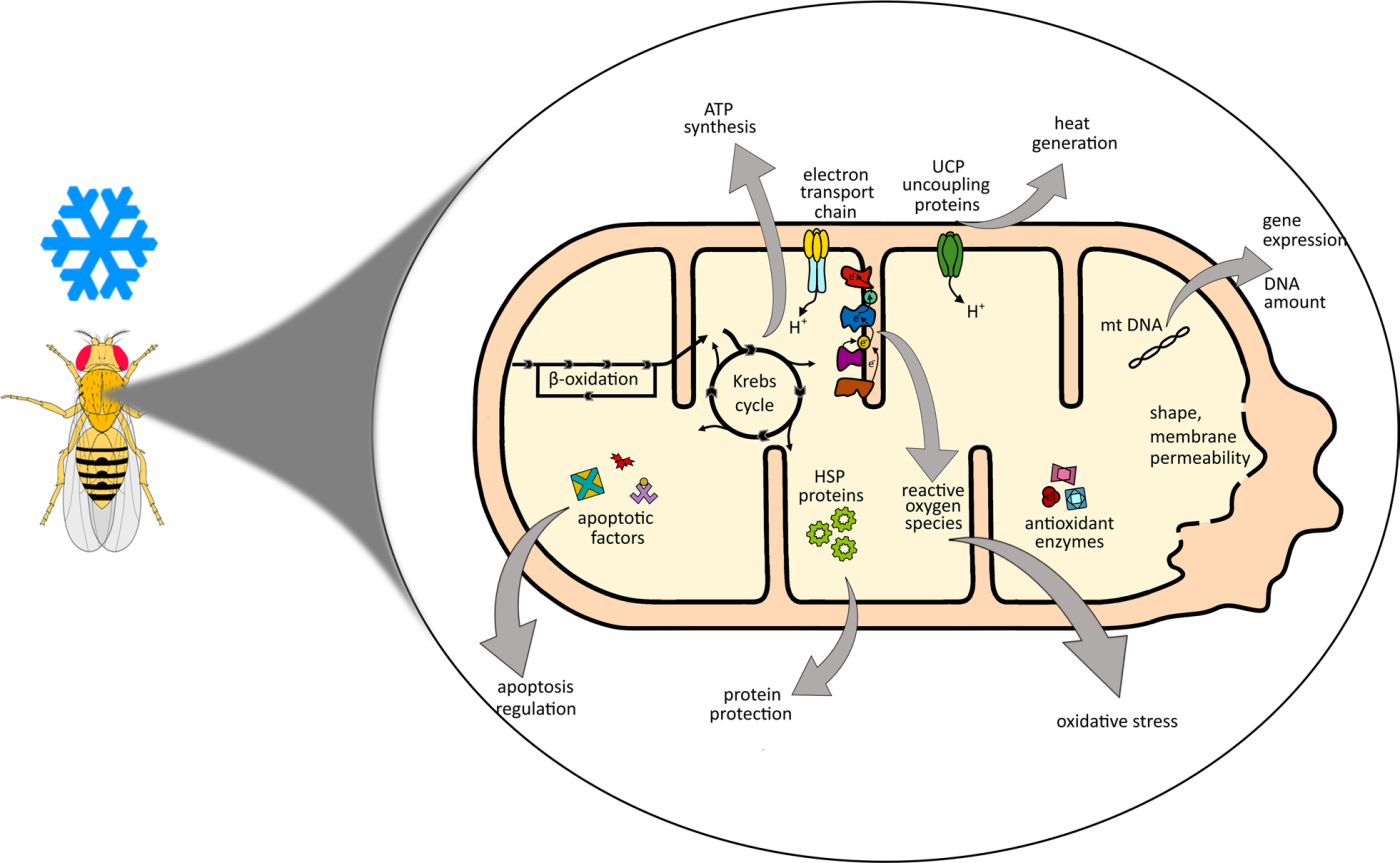

## Introduction

**Fig. 1:**
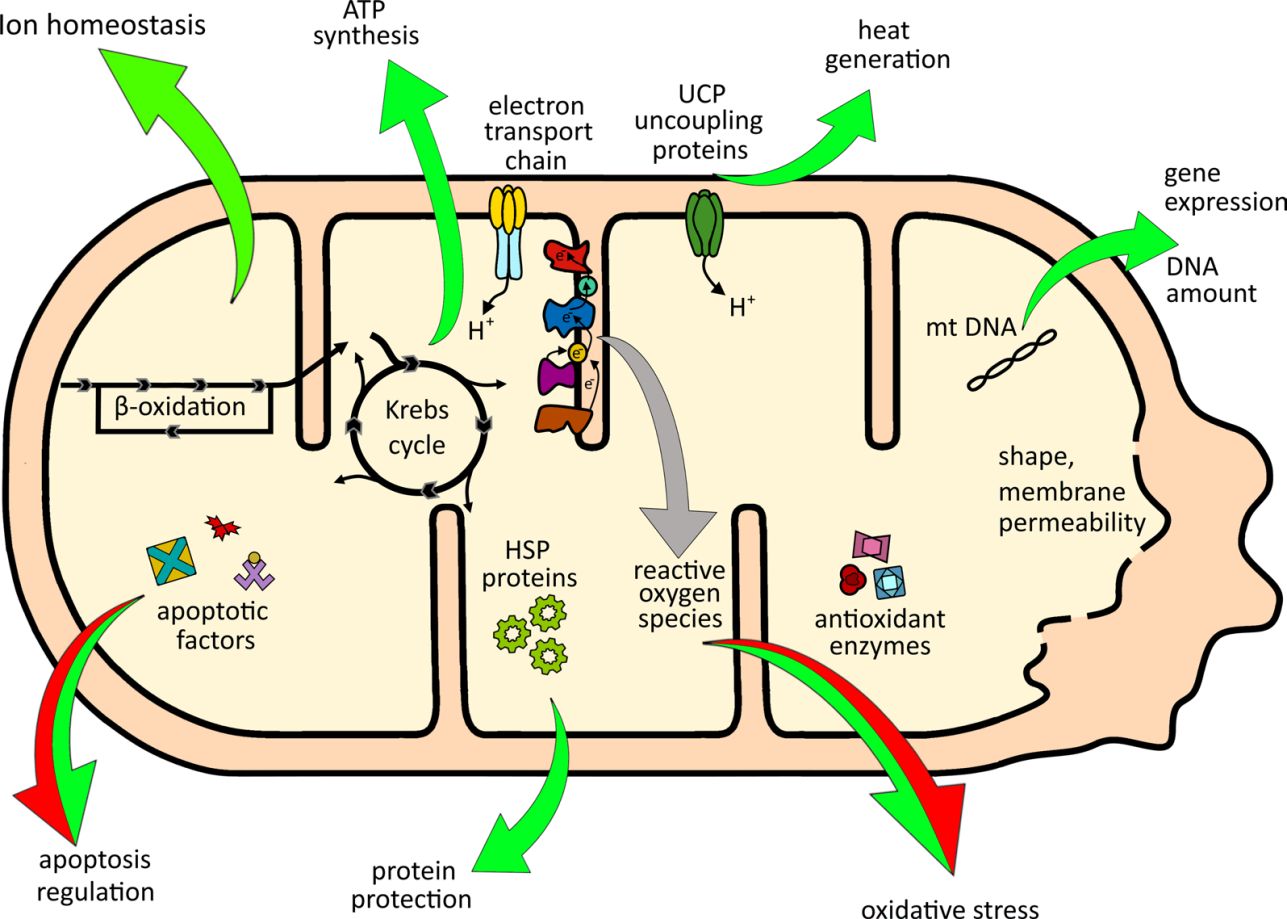
The multi-directional role of mitochondria during cold stress. The mitochondria are both the target of cold stress and the central hub that coordinates the cell response to it. During cold stress, the energy metabolism of these organelles changes in order to maintain the functionality of the entire cell. Insects faced with cold stress change their cellular metabolism, which often results in increased accumulation of ROS by mitochondria. Consequently, the mitochondrial antioxidant system during cold stress is modulated to counteract the negative effects of cold-associated ROS formation [37]. It is also increasingly obvious that the mitochondrial integrity and cellular signaling associated with mitochondria are essential for sustaining ion and energetic homeostasis of the cell and its survival [28]. Tight regulation of apoptosis by mitochondrial pathway is essential for survival as the stimulated activity of caspases is not solely the indicator of apoptosis but besides, it demonstrates nonapoptotic functions i.e., control of a cell shape, cell migration or proliferation. Mitochondria may also take part in heat dissipation which is caused by uncoupling of the respiration by these organelles [55]. If these mechanisms fail to adapt the cell to prevail the cold stress and the accumulation of cold-injuries is increasing, the mitochondria commence the processes leading to the programmed cell death pathway via apoptosis to utilize and recycle damaged cells and their components [38]. Green arrows indicate mitochondria regulated processes leading to the cell survival pathway, whereas red arrows processes leading to cell death

All types of cells are active energetic units that need a constant supply of energy. In animal cells “power plants” which provide it are mitochondria. Mitochondria are a double membrane-bound organelle found in most eukaryotic organisms which provide energy from substances accumulated in food, ending in the production of a prominent energy carrier of the cell, ATP. According to endosymbiotic hypothesis, theses organelles were originally prokaryotic cells that became endosymbionts living inside the eukaryotes, implementing oxidative mechanisms generating energy [1]. However, this is not their only role, as mitochondria are associated with almost all major aspects of eukaryotic evolution from aging, senescence to reproduction and immune response [2–6]. They function as a cellular hub that links metabolism, stress sensing, signaling, and survival of the cell [5, 7, 8] (Fig. 1). Hence, it is not particularly surprising that mitochondria can play a role in environmental adaptations [3, 9, 10].

Insects are constantly challenged by unfavorable environmental conditions such as pathogen infections, UV radiation, the action of insecticides, oxidative stress, and temperature—either high or low [11–15]. Temperature stress is without a doubt the most critical factor among abiotic stressors affecting the physiology of insects. Extreme temperatures or large temperature fluctuations can be harmful to ectothermic organisms either through direct effects, which include temperature-induced cellular or tissue injury and/or through indirect effects, e.g., limitations in their activity [16–20].

Maintaining cellular energy homeostasis is a challenge for insects, which are exposed to low stressful temperatures in nature. During their lifetimes, insects have the capacity to adjust their physiological mechanisms to promote cold tolerance and cope with sublethal thermal conditions, a phenomenon referred to as thermal acclimation [21–26]. Based on the ability to withstand and survive freeze stress (temperatures where its body fluids might be expected to freeze), insects fall into the following categories.

1. *Chill susceptible* insects die from cold-induced injuries before the formation of ice occurs within their bodies [27–29]. This strategy is sometimes referred to as cold-intolerance. For example, the larvae of the false codling moth, *Thaumatotobia leucotreta* (Lepidoptera: Tortricidae), freeze between − 13 and − 22 °C but are killed by brief exposures between − 8 and − 12 °C [30]. The cold tolerance of these chill-susceptible insects is therefore determined by their ability to survive exposure to temperatures above their freezing point [29, 31, 32]. Vast majority of insects fall into this category as they are not able to handle severe sub-zero temperatures (for details see Overgaard et al. [28], Overgaard and MacMillan [27] and references within). More recently, species falling to this category have been further classified into chill-susceptible or chill-tolerant species, depending on their relative sensitivity to low temperature [27].
2. The second strategy is *freeze-avoidance*. Species utilizing this strategy are capable of surviving low temperatures by lowering their freezing point of extracellular compartments of their body by supercooling via accumulation of cryoprotectants. For example, prepupae of the emerald ash borer, *Agrilus planipennis* can survive prolonged exposures to subzero temperatures, provided they do not freeze, and in the winter have SCPs (Supercooling point; the temperature at which spontaneous ice formation is initiated in the insects body fluids) below − 25 °C [33].
3. Lastly, there are examples of insects that can tolerate and survive freezing of their tissues and body—*freeze-tolerant* ones. The most profound example of insect using this strategy is the fly *Chymomyza costata*, which is capable of surviving immersion in liquid nitrogen [34].

Regardless of the strategy used, in some insects also occur the phenomenon of *diapause*. Perfect examples are two species of goldenrod gall insects, *Epiblema scudderiana* and *Eurosta solidaginis*, the first being freeze-avoiding and the second being a freeze-tolerant species. Yet, for both winter survival includes entry into diapause [35]. Diapause is an adaptation assisting in survival of insects through periods of harsh environmental conditions and adverse seasons. Many insects distributed in the temperate zones enter winter diapause to survive seasonal environmental stresses. The term diapause does not necessarily imply cold hardiness. It is a genetically determined and an endocrine-mediated dormancy that occurs at a specific developmental stage according to the insect species [36]. Examples of egg, larval, pupal, and adult diapause are all well known, but, for most species, the genetic capacity for diapause is restricted to only one of these stages. In diapause, the metabolic rate characteristically drops far below the rates observed in nondiapausing individuals. Food intake is reduced, behavioral changes are frequently observed and need for mitochondrial activity is diminished [35, 36].

The activity and integrity of mitochondria without a doubt plays a crucial role in response to and overcoming cold stress. While the complexity of the mitochondrial bioenergetic and signaling network is not yet fully understood, it is increasingly evident that maintenance of the mitochondrial integrity and mitochondrially-derived cellular signaling are critical for cellular homeostasis and survival [7, 37, 38]. They are both the target of cold stress and the central hub that coordinates the cell response to it. Numerous eukaryotes, insects among others, can survive and maintain mitochondrial homeostasis despite the frequent and drastic fluctuations in environmental conditions. Studies on marine ectotherms have shown that oxygen consumption by mitochondria is a good indicator of thermal adaptation and acclimation [39]. However, studies on insects indicate varied effects of cold stress on energy production. Depending on species, strategy of cold resistance and “type of cold stress”, that effects might concern changes in ATP level and mitochondria driven energetic homeostasis, which leads consequently to oxidative stress if the production of reactive oxygen species (ROS) overcomes the antioxidative response capabilities of these organelles. Cold stress affects also their morphology, and abundance; as well as mitochondrial and nuclear gene expression pattern, including genes encoding mitochondrial energetic machinery proteins or heat shock proteins (HSP) which act to protect and deal with the effects of stress [40–56]. It is also growingly obvious that the mitochondrial integrity and cellular signaling associated with mitochondria are essential for sustaining the ion and energetic homeostasis of the cell and its survival. As was shown insect mitochondria may also take part in heat dissipation by uncoupling the respiration to protect the cell from harmful effects of cold [54, 57]. If these adaptive mechanisms are inefficient to overcome the cold-stress and the cell damage accumulates the mitochondria commence the processes leading to the programmed cell death pathway as its regulators [58] via apoptosis, to dispose damaged cells [38]. Moreover, what should be kept in mind is that the role of mitochondria in response to cold stress as well as the direction of changes in their activity might vary depending on the species and in what climatic zone they naturally occur, and whether they were acclimated or not to the cold stress. Differences in response to cold stress depending on aforementioned are shown in Table 1. Nevertheless, taking all of that into consideration, we believe that insects can be categorized into three distinct categories implicating mitochondria in cold stress survival. (1) First are historically most studied, chill susceptible insects. Recent studies have shown that it is the ability to maintain active transport that allows these insects to overcome cold stress (reviewed in Overgaard and MacMillan [27], Overgaard et al. [28]). Cold stress will potentially lower the ATP production in aerobic metabolism, thus limiting active transport below passive flux. Hence chill susceptible insects by adaptation or acclimation will ensure mitochondrial function for ATP production. The ability to do this most probably define the limit of cold tolerance. (2) Insects entering the diapause, undergo metabolic depression resulting in re-setting of metabolism. Reduction of aerobic ATP production is accompanied by shift in substrate metabolism to allow for accumulation of cryoprotectants. These species will adjust ATP capacity to allow for such shifts. (3) Lastly, there are the “extreme survivals”. Insects capable of surviving extreme freezing temperatures—including freezing of the ECM which leads to cell and organelle dehydration.

**Table 1:** Changes in genes, energetic parameters of mitochondria and proteins in chill susceptible/chill tolerant or freeze avoiding/tolerant insects after cold/freeze stress

			Chill susceptible/Chill tolerant		Freeze avoiding/Freeze tolerant
Genes	Acclimated	↑	*ATPase* (*A.* *colemani*) [100]^b^	↑↓	*COX* (*E*. *scudderiana*) [35]^c^
				↑↓	*12S rRNA* (*E*. *scudderiana*) [35]^c^
				↑	*HSP60* (*D. antiqua*) [103]^c^
				↓	*HSP60 (L. decemlineata*) [104]^c^
	Non-acclimated	↑	*COX* (*D. simulans*) [51]	↑	*HSP60* (*B. antarctica*) [53, 105]
		↑↓	*HSP60* (*D. melanogaster*) [106]	↑	*HSP60* (*F. occidentalis*) [107]
		↑	*HSP22* (*D. melanogaster*) [106]	↑	*HSP60* (*G. daurica*) [108]
		↑	*HSP23* (*D. melanogaster*) [106]	↑	*HSP60* (*M. alternatus*) [109]
		↑	*mt:ND4* (*D. melanogaster*) [52]		
		↑	*mt:ND5* (*D. melanogaster*) [52]		
		↑↓	*mt:COXI* (*D. melanogaster*) [52]		
		↑↓	*mt:COXII* (*D. melanogaster*) [52]		
		↑	*UCP4C* (*D. melanogaster*) [54]		
		↑↓	*HSP23* (*D. melanogaster*) [110]		
		↑	*HSP23* (*S. crassipalpis*) [111]		
		↑	*AccSCO2* (*A. cerana*) [112]		
		↑	*MUP2* (*A. melifera*) [55]		
Energetic parameters	Acclimated	↑	ATP (*A. diaperinus*) [41]^b^	↓	cyt. b (*G. groenlandicai*) [84]
		↑	TCA (*A. colemani*) [61]	↓	3-hydroxyacyl-CoA dehydrogenase (*E*. *solidaginis*) [79]^c^
		↑	citrate (*D. melanogaster*) [77]	↓	thiolase (*E*. *solidaginis*) [79]^c^
		↑	aconitate (*D. melanogaster*) [77]	↓	cyt. c (*G. groenlandicai*) [44]
		↓	ketoglutarate (*D. melanogaster*) [77]	↓	CS (*C. costata*) [76]
		↓	succinate (*D. melanogaster*) [77]	↓	NAD-IDH (*E*. *solidaginis*) [47]^c^
		↓	fumarate (*D. melanogaster*) [77]	↓↑	state 4 (*C. costata*) [59]^c^
		↓	OCR (*D. melanogaster*) [60]	↑	ATP (*C. costata*) [59]^c^
		↑	ADP/O (*D. melanogaster*) [60]^a^	↑	3-hydroxyacyl-CoA dehydrogenase (*E.* *scudderiana)* [79]^c^
		↑↓	RCR (*D. melanogaster*) [60]	↑	thiolase (*E.* *scudderiana)* [79]^c^
		↓	ATP (*D. melanogaster*) [60]		
		↑	ATP (*S. crassipalpis*) [44]^b^		
	Non-acclimated	↓	RCR (*G. coquereliana*) [89]	↑	NAD-IDH (*E*. *solidaginis*) [47]^c^
		↓	state 3 (*G. coquereliana*) [89]	↓	State 4 (*C. costata*) [59]^c^
		↓	OCR (*D. melanogaster*) [60]	↓	GDH (*E*. *scudderiana*) [47]^c^
		↑↓	RCR (*D. melanogaster*) [60]		
		↓	ATP (*D. melanogaster*) [60]		
		↓	ATP (*S. crassipalpis*) [44]		
Proteins, enzymes	Acclimated	↑	Aconitase (*A. colemani*) [61]	↓	COX (*E*. *solidaginis*) [47]^c^
		↑	Fumarase (*A. colemani*) [61]	↑	HSP70 (*E. solidaginis*) [113]^b,^^c^
		↑	Malate dehydrogenase (*A. colemani*) [61]	↑↓	HSP70 (*E. solidaginis*) [113]^c^
				↓	COX (*E*. *scudderiana*) [35, 47]^c^
				↑↓	SOD (*E*. *solidaginis*) [114]^c^
				↑	SOD (*E.* *scudderiana)* [114]^c^
	Non-acclimated	↓	GSHt (*A. diaperinus*) [115]		
		↑	UCP (*G. coquereliana*) [89]		
		↑	HSP70 (*G. coquereliana*) [89, 116]		
		↑	HSP70 (*S. exigua*) [117]		
		↓	HSP60 (*S. exigua*) [117]		
		↓↑	procaspase-9-like (*D. melanogaster*) [58]		
		↓↑	caspase-3-like (*D. melanogaster*) [58]		
		↓	Bcl-2 (*D. melanogaster*) [58]		

The table is divided also into research on insects acclimated to low temperatures (acclimated) and insects not acclimated to low temperatures (non-acclimated). Empty cells in the table indicate that research in this direction has not been conducted and much is still to unravel. As each division would be artificial, we decided to rank insects into species in which cold-induced injury, and death occurs at temperatures above temperature causing extracellular freezing (Chill susceptible/Chill tolerant) and species in which injury is related to ice formation (Freeze avoiding/Freeze tolerant) (for review see Overgaard and MacMillan [27]). Arrows indicate: ↑ increase/up-regulation, ↓ decrease/down-regulation, ↑↓ no change

^a^Compared to non-acclimated counter partners

^b^FTR or freeze–thaw cycles were used in the studies and not cold-acclimation per se

^c^Diapausing insects

Since the mitochondrial bioenergetic and signaling network is very complex and much is still to unravel to fully understand it, it is more obvious that maintenance of the mitochondrial function and mitochondrially-derived cellular signaling are critical for cellular homeostasis and survival. This indicates that mitochondria appear to be an interesting target for research regarding cold stress tolerance in insects, and the knowledge about their role in this process can be deeply explored in the future. Hence, this review summarizes the existing knowledge on the role of the cell’s “power plants” and their role in insects' resistance to cold stress, indicating new potential research perspectives.

## Energetic homeostasis

A key aspect of the response to cold stress is the modulation of major metabolic pathways responsible for energy metabolism. Oxygen-based respiration offers an enormous advantage for cellular energetics. The production of ATP by complete oxidation of substrate provides more energy in form useful for cells than anaerobic processes. And because the energy is at the core of all life processes, thus, the cells’ ability to produce energy will undergo significant changes depending on cold stress conditions and species-related strategies. The changes will be expressed on different levels from genes through proteins to whole organelles activity and will depend on duration of temperature exposure, temperature altitude, latitude, or/and organismal acclimation to cold stress [56, 59, 60].

### Glycolysis

Glycolysis is the central pathway for the glucose catabolism in which glucose is converted into pyruvates. Given the importance of nutrient flow through this central metabolic pathway as the source of ATP and production of building blocks for many synthesis reactions, adaptation to thermal stress is expected to modify this pathway. Colinet et al. [61] studied proteins that responded to constant cold or a fluctuating thermal regime (FTR) in a chill susceptible parasitic wasp *Aphidius colemani*. Proteins that were upregulated in response to FTR included several associated with glycolysis (i.e. aldolase, phosphoglycerate kinase or glyceraldehyde-3-phosphate dehydrogenase. Using pyrosequencing Hahn et al. [62] studied *Sarcophaga crassipalpis* to identify diapause-responsive genes and compare the gene responses of flesh fly pupae with those of adult diapause in *D. melanogaster*. They found enhanced expression of genes associated with glycolysis and gluconeogenesis (i.e. hexokinase, aldolase, phosphofructokinase) [62, 63]. Later in 2016 Williams et al. [64] also provided evidence for increased reliance on glycolysis in *Drosophila melanogaster* during cold stress. In another drosophilid, *D. suzukii* it was found that winter-acclimated individuals also exhibit an up-regulation of glycolysis genes [65]. In diapausing flesh fly *S. crassipalpis* Michaud and Denlinger [66] demonstrated, using metabolomic approach an increase in the glycerol, glucose, alanine, pyruvate—metabolites involved in glycolysis. Together with depression of tricarboxylic acid (TCA) cycle (discussed later on) this could promote the synthesis of glycerol, the major cryoprotectant in insects [67, 68]. Since glucose carbons may be converted into a number of different compounds through the glycolytic pathway and its branches [66], activation of glycolysis pathway may also promote the increase in the concentration of sugars, polyols or free amino acids which all may act as low-molecular weight cryoprotectants [69–71]. Cryoprotectants lower the melting and supercooling points in chill tolerant and freeze-avoiding insects and in freeze-tolerant ones, they lower the proportion of ice [72–74].

Furthermore, as evidenced by glycolytic end product (lactate, alanine) accumulation during freezing, freeze-tolerant species *E. soldiganis* rely on anaerobic metabolism while frozen [50]. This is supported by an observed transient elevation of glucose-6-P levels, suggesting a mobilization of glycogen as fuel, in the early hours after freezing and by continuous accumulation of lactate throughout the long-term freezing exposure [75]. Similar results were obtained in diapausing freeze-tolerant *C. costata* [76]. In stressed insects (supercooled, freeze-stressed or cryopreserved in liquid nitrogen) were observed symptoms of anaerobic metabolism evidenced by high levels of lactate, succinate, and alanine. Although insects from each treatment showed high level of survival, the larvae preserved in liquid nitrogen showed impaired mitochondrial function demonstrated by the accumulations of glucose and several derivates of the glycolytic pathway side-branches (fructose, myoinositol, sorbitol, and glycine). This suggests that the glycolytic flux is partially diverted from Krebs cycle and production of energy toward accumulation of alanine [76].

### Krebs cycle

Up-regulation of proteins playing crucial roles in TCA cycle was observed in chill susceptible parasitic wasp *A. colemani* [61]. The elevation of Krebs enzymes level was associated with reduced mortality under fluctuating thermal regimes (FTR), when insect exposure to cold was interrupted daily by a transfer to 20 °C for 2 h, compared to constant low temperature (CLT). Expression of genes encoding aconitase, fumarase and malate dehydrogenase increased during FTR, which may come along with the increase in ATP demand, and thus playing a protective role in maintaining of the energy balance [61]. Metabolite changes were also observed in *D. melanogaster* larvae under CLT and FTR conditions [77]. Some of the intermediates of TCA, citrate and aconitate, increased, whereas the others: ketoglutarate, succinate, fumarate, and malate decreased suggesting a slowdown of intermediary metabolism, including TCA turnover by a blockade of isocitrate dehydrogenase, which converts isocitrate to ketoglutarate. The changes were faster under CLT as for FTR. Activities of citrate synthase, NAD-isocitrate dehydrogenase and glutamate dehydrogenase were reduced more than 50% during the winter months in larvae of goldenrod gall insects, freeze-avoiding *E. scudderiana* and freeze-tolerant *E. solidaginis* [47, 48]. In these insect species, a strong decrease of the activity in rate-limiting enzyme for carbohydrate oxidation, pyruvate dehydrogenase complex (PDC) (by around 80% and 50%, in *E. scudderiana* and *E. solidaginis,* respectively) was also observed [48]. The activity of citrate synthase (CS), a marker of mitochondrial integrity as well as oxidative capacity, was 1.5-fold lower in fat body mitochondria of diapausing, compared to active (non-diapausing) larvae of *C. costata* [76]. Lethally frozen non-diapausing flies showed relatively slight decrease in CS activity but almost total loss of respiration capacity. No changes in CS activity with maintained capacity for oxygen consumption was observed upon freezing either in diapausing insects and non-diapausing fed with proline-supplemented diet [59]. In this last case, proline and other cryoprotective substances may be a part of adaptative changes. It was shown in CS from an Antarctic bacterial strain DS2-3R that the presence of proline residues in α helices changes the flexibility of the enzyme which in turn enables the catalytic activity of the protein at lower temperatures [78]. Perhaps similar properties can be observed in mitochondria of cold-resistant insects, hence crystallographic studies in this filed could be of great value. In the fat body of cold stressed larvae, the silkworm *Philosamia ricini* increased activity of malate dehydrogenase (MDH) and lactate dehydrogenase (LDH) was consistent with an augmented anaerobic metabolism leading to ethanol, lactate, and alanine as end-products. Reorganization of metabolic activity observed by accumulation of glycolytic products, lactate and alanine during freezing in *E. solidaginis* suggests that anaerobic respiration might be one of the important processes for energy supply over the winter in freeze-tolerant insects [49]. On the other hand, no evidence for disruption of aerobic metabolism (e.g., no lactate accumulation) in freeze-avoiding species *E. scudderiana* was observed.

### Fatty acids oxidation

In contrast to Krebs cycle enzymes, activities of mitochondrial enzymes involved in fatty acid oxidation (3-hydroxyacyl-CoA dehydrogenase, thiolase) increased two- to fourfold over the winter in freeze-avoiding *E.* *scudderiana* [79], suggesting that aerobic oxidation of lipids in freeze-avoiding insects supports metabolic requirements during the period of low temperatures what indicates for the pivotal role of lipid metabolism in these insect species during cold. Different strategy upon cold was observed in freeze-tolerant *E. solidaginis*, where the activity of enzymes engaged in fatty acid synthesis (malic enzyme) and fatty acids oxidation (carnitine palmityol transferase, 3-hydroxyacyl-dehydrogenase CoA or thiolase) were suppressed [79].

### Respiratory chain and oxidative phosphorylation

Mitochondrial enzymes and proteins of respiratory chain have been shown to be suppressed during the winter months in larvae of goldenrod gall insects freeze-avoiding *E. scudderiana* and freeze-tolerant *E. solidaginis* [47]. In these species, a decrease of cytochrome c oxidase (COX) activity has been observed. On contrary, chill susceptible fly *Drosophila simulans*, had higher cytochrome c oxidase activity than their more resistant counterparts [51]. COX, located on the inner mitochondrial membrane, is the terminal electron acceptor in the respiratory chain responsible for the reduction of dioxygen to water and one of the three sites of proton pumping across the inner membrane that creates the electrochemical gradient that drives oxidative phosphorylation [80, 81]. The defects in the assembly and function of COX are frequent causes of oxidative phosphorylation disorders, which primarily affect organs with high energy demands [82, 83]. In freeze-avoiding *E. scuderriana*, COX activity decreased about one-third, however at the same time *COX1* mRNA transcripts and *12S rRNA* levels were unchanged as was evidenced for *COX1* DNA content [35]. Ballard et al. [51] observed that in *D. simulans* variations in mtDNA are associated with differences in cytochrome c oxidase activity. Significantly lower activity of cytochrome c oxidase was determined for flies harboring *s*/II than *s*/III mtDNA lines. The differences in mtDNA sequence of fly lines were correlated with a serine replacement in COXII. Those authors showed that flies harboring *s*/II mtDNA are more cold-tolerant. Looking at the genes encoding respiratory chain proteins, Camus et al. [52] showed that differences in mtDNA resulted in varied thermal sensitivity of *D. melanogaster.* They found two haplotypes (A1 and B1), depending on whether the insects were from tropical or temperate origins, differing from each other by 15 single nucleotide polymorphisms (SNPs). Flies harboring the B1 haplotype with higher level of gene expression of *mt:ND4* and *mt:ND5* are superior at withstanding an extreme cold challenge, relative to their A1 counterparts, proving that thermal tolerance phenotypes are directly correlated with the mtDNA sequence. The authors examined the expression patterns of five genes involved in complex I (NADH dehydrogenase) and complex IV (cytochrome oxidase) of the electron transport chain (complex I: *mt:ND4*,* mt:ND5*, complex IV: *mt:COXI*, *mt:COXII*, *mt:COXIII*). They found that flies bearing the B1 haplotype had a higher level of gene expression of *mt:ND4* and *mt:ND5*, showing that genetic variation within complex I may affect the thermal tolerance [52] (Fig. 1). Interestingly, Camus et al. [52] did not observe any changes in gene expression of complex IV genes, such as *mt:COXI,* or *mt:COXII.* Moreover, cold stress may affect fat body cytochrome systems, mainly cyt b and cyt c which are crucial in electron transport through the respiratory chain. In frozen and cold-acclimated *Gynaephora groenlandica* larvae, the content of these cytochromes was slightly reduced compared to control insects [84].

The winter drop-in COX activity, as well as other mitochondrial enzymes, is probably a consequence of the suppression of protein synthesis to reduce the amounts of selected enzymes in mitochondria during cold exposure (slowdown of metabolic activity), or inhibition of enzyme activities, perhaps by reversible controls [80]. Lowered enzyme activities could be also an effect of decreased numbers of mitochondria and suppression of mitochondrial biogenesis. The changes in activity of enzymes and no fluctuations in mtDNA content as well as transcript levels of mitochondrially encoded genes, *COX 1* and *12S rRNA* may suggest that changes in enzymatic activity of COX are likely mediated via post-translational modification or allosteric regulation [35].

Electron transport through respiratory chain is the main pathway responsible for ATP synthesis, which is required for activities of ion pumps and HSPs or synthesis of cryoprotectants (e.g., glycerol) [41, 85]. Environmental stressors such as cold may disturb energy homeostasis, leading to dysfunction of mitochondrial physiology reflected in decreased coupling and ATP production as well as cellular chilling injuries e.g., membrane alteration and loss of ion or water homeostasis [86–88]. However, the knowledge about the disruption of cellular bioenergetics in insects exposed to low temperature stress, regarding mitochondria is still very scanty. So far, only two studies on mitochondria isolated from cold-stressed insects were investigated by Colinet et al. [60] and Chowański et al. [89]. These researchers analyzed mitochondrial condition by determining of oxygen consumption rates (OCRs) in resting state 4 respiration, phosphorylating state 3 (after ADP addition), and the degree of mitochondrial coupling reflected in respiratory control ratio (RCR). This parameter is the most useful measure of isolated mitochondria function and reflects the adjusting the respiration rate (electron transport) to the actual demand for ATP. ADP/O ratio, indicates efficiency of phosphorylation in mitochondria (ATP synthesis) measured as the amount of oxygen uptake stimulated by an addition of a known amount of ADP [90].

**Fig. 2:**
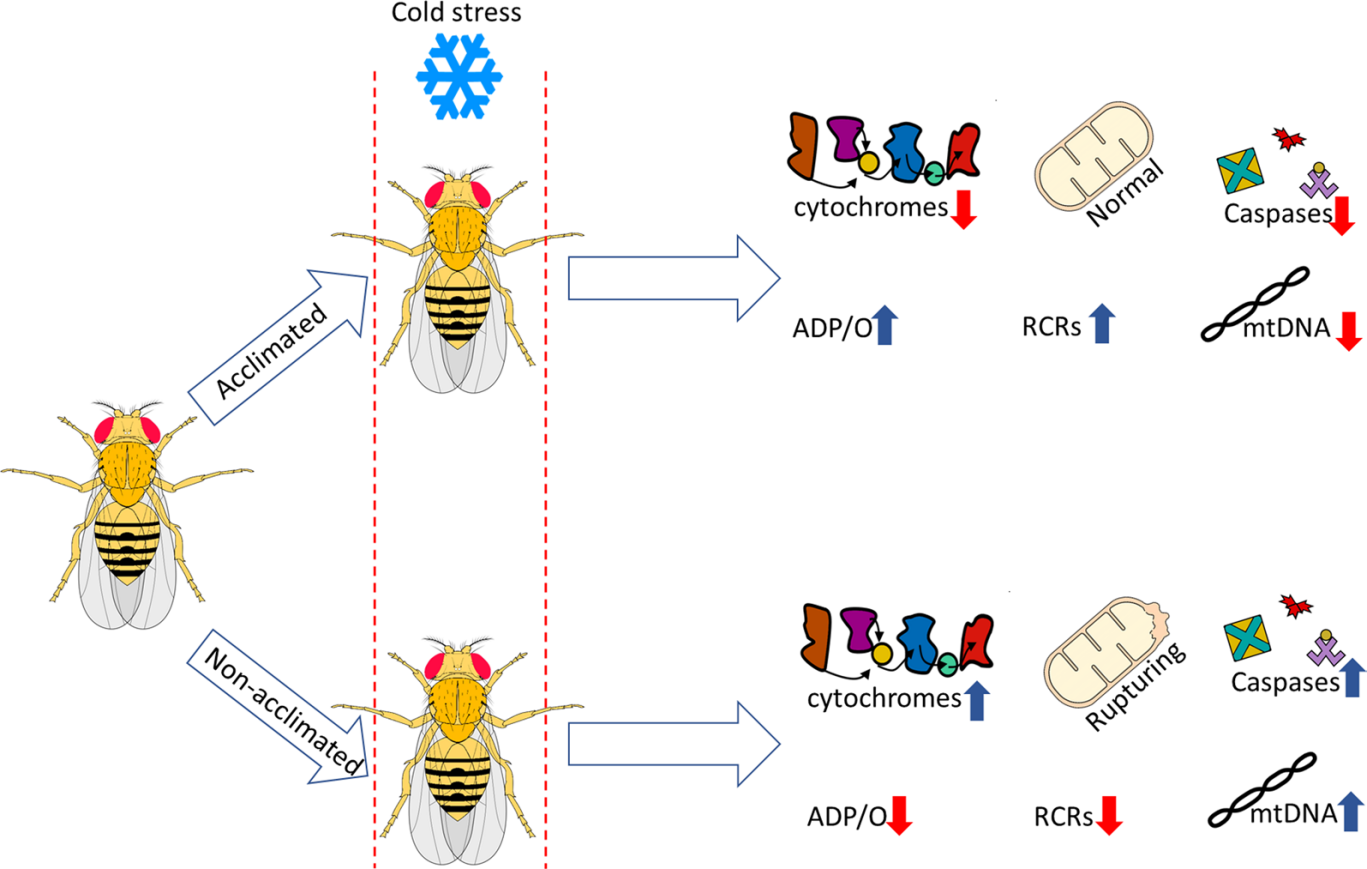
Scheme representing the effects of cold stress on mitochondria of cold-acclimated and non-acclimated insects. Arrows indicate changes (red—decrease and blue—increase) in particular parameters of insects compared to their counterparts (acclimated vs non-acclimated). ADP/O—mitochondrial phosphorylation efficiency, RCR—respiratory control ratio reflecting mitochondrial coupling

Colinet et al. [60] monitored ATP synthesis in *D. melanogaster* flies exposed to chronic cold stress of 4 °C in cold-susceptible (CO) and cold-acclimated (CA) phenotypes. In all cases, OCRs and ATP production were significantly affected by the time of cold exposure and decreased with increasing duration of cold stress. RCRs and ADP/O ratios were not influenced by the time of cold exposure, but ATP production rates and ADP/O ratio were affected by temperature and were higher when mitochondria were assayed at 25 °C versus 4 °C. The efficiency of oxidative phosphorylation depended on cold acclimation and was higher in mitochondria from CA compared to CO flies (Fig. 2). Oxygen consumption rates were independent of acclimation treatment, although RCRs were elevated in CA *vs.* CO flies. Like in other ectotherms [91], higher RCR in CA flies primarily reflected higher mitochondrial oxidative phosphorylation activity (State 3), whereas resting respiration (State 4) remained identical in both phenotypes. On the other hand, respiration measurements in larvae of fly *C. costata* showed that freeze-sensitive (non-diapausing) phenotypes exhibited decreased oxygen consumption rates, whereas mitochondria of freeze-tolerant insects (diapausing, cold acclimated) maintained respiratory capacity during cold stress [59].

The decline in ATP synthesis in control insects after prolonged cold exposure was associated with reduced survival capacity. In contrast, cold-acclimated flies exhibited high survival and maintained higher rates of mitochondrial ATP synthesis and coupling. Concluding, cold acclimation increases rates of ATP, mitochondrial coupling and survival of insects, thereby by maintaining of bioenergetic homeostasis improves their cold tolerance. In chill tolerant tropical cockroach *Gromphadorhina coquereliana* exposed to 3 h of cold stress at temperature 4 °C, mitochondria isolated from leg muscle and fat body tissue exhibited decreased RCR, indicating a lower mitochondrial coupling [89]. In the fat body mitochondria, the drop in RCR ratio was accompanied by the decrease in the rate of phosphorylation whereas in muscle mitochondria state 3 phosphorylation remained unchanged. In other chill susceptible insect, tropical lesser mealworm *Alphitobius diaperinus* thermal regime, constant or fluctuating disturbed ATP supply, however, periodic short pulses of warming (for recovery processes) were enough to restore ATP homeostasis and improve cold survival [41, 92]. Higher levels of ATP may suggest that respiratory function of mitochondrial enzymes in *A. diaperinus* beetle was not critically affected by cold, and ATP synthesis exceeds ATP consumption. The drop in ATP levels was observed in chill tolerant flesh flies *Sarcophaga crassipalpis* exposed to constant 0 °C for 20 days and it was associated with high mortality (up to 99%). However high temperature pulses elevated ATP levels and decreased indirect injury what reflects in higher survival [44].

The disruption of ATP pool in insects exposed to low temperature stress may be explained by changes in enzymatic activity of respiratory chain complexes or/and structure and function of mitochondrial membranes. The alterations in permeability of biological membranes and activity of membrane-bound enzymes, such as Na^+^/K^+^-ATPase under cold exposure was already reported by MacMillan et al. [86], Kostal et al. [87], and Cheslock et al. [93]. Altered permeability of cellular membranes leads to loss of ion/water homeostasis [94, 95] and membrane depolarization [96, 97], whereas inhibition of Na^+^/K^+^-ATPase negatively affects mitochondrial bioenergetics [98]. Štětina et al. [59] showed that adaptive changes, such as protection of the inner mitochondrial membrane against permeability transition and mitochondrial swelling in larvae of fly *C. costata*, could be related to the phenotypic transition to cold acclimation or diapause. Biological membranes, including the inner mitochondrial membrane (IMM) in freeze sensitive insects (non-diapause, warm-acclimated), are susceptible to freezing stress (− 5 °C and − 30 °C), which may cause loss of barrier function, an osmotic influx of cytosolic water into the matrix, and consequently, mitochondrial swelling (Fig. 2). In this case respiration analysis of *C. costata* fat body mitochondria revealed significant drop in oxygen consumption. In contrast, freeze-tolerant larvae of *C. costata* (either diapause, cold acclimated or non-diapause proline-augmented diet fed) exhibited no mitochondrial swelling and no loss of respiration capacity [59].

Research carried out so far indicates that response to cold stress in insects is tissue- and species-specific. However, some parameters such as disruption of ATP synthesis seem similar either in tropical insects (*Gromphadorhina* *coquereliana*) or insects from the temperate zones (*D. melanogaster*) exposed to low temperature. The important process of insect’s acclimation to cold modifies the efficiency of oxidative phosphorylation which is higher in mitochondria from CA compared to CO insects and finally promotes cold tolerance [41]. Changes due to cold acclimation are reflected not only by maintaining mitochondrial bioenergetics capacity but in the whole organism because of an extensive biological reorganization, almost one-third of the transcriptome and nearly half of the metabolome [41, 99]. For example, upregulation of ATP-synthase, a multisubunit enzyme of inner mitochondrial membranes responsible for the generation of ATP [100] has been found under FTR in *A.* *colemani.*

In some cases, oxidative phosphorylation efficiency can be decreased by activation of uncoupling proteins (UCPs), integral proteins of the inner mitochondrial membrane [101]. UCPs activity is stimulated by free fatty acids (FFA) and inhibited by purine nucleotides (PN). For the first time in insects, UCPs have been functionally characterized in cockroach *G.* *coquereliana* [102]. Changes in GcUCP4 activity observed in the fat body and leg muscle of this tropical insect during cold exposure indicate engagement of UCPs in the response to cold stress [89]. It was evidenced the main role of UCPs is diminishing of oxidative stress (described in the *Oxidative stress* paragraph), although their other function in insects cannot be excluded. In brown adipose tissue of hibernating mammals, uncoupling is the crucial mechanism responsible for heat generation. Similarly, the higher activity of UCP4 in the fat body of *G.* *coquereliana* cockroach may indicate the thermogenic role of UCPs (and fat body tissue) in cold stressed insects (Fig. 1). The fat body is a cytological analogue of brown adipose tissue and functional analogue of mammalian liver. The possible involvement of UCP4 in heat generation by uncoupled respiration has been previously suggested by Da-Re et al. [54] and Ramirez et al. [55]. In *D. melanogaster* larvae, by silencing *Ucp4C*, the authors demonstrated that it is essential for larval development at low temperatures. Ramirez et al. [55] showed that chill susceptible *Apis mellifera* brood transferred from the control temperature of 36 °C to colder conditions of 25 °C strongly increased the transcript level of uncoupling protein (*MUP2*). In parallel, the ATP level tends to diminish in the cold-stressed brood. These results suggest a potential thermogenic role of MUP2 in honeybee brood reared at low temperatures. In recent study, Ulgherait et al. [57] demonstrated in *D. melanogaster per*^*01*^ mutants, in which the expression of *Ucp4B* and *Ucp4C* is constitutively high, that after 1 h of cold shock at 4 °C, the flies recover significantly quicker than control specimens. Suggesting that *per*^*01*^ mutants may generate more heat than controls. In addition, disruption of *Ucp4B/C* in *per*^*01*^ flies reverted whole-animal cold shock recovery rates. When *Ucp4C* is overexpressed in wild type flies, they show faster cold shock recovery as the *per*^*01*^ mutants [57]. This indicates that UCPs take part in cold stress response probably by heat dissipation. However, further studies on plausible thermogenic function of UCPs in insects are required.

Considering above facts, we can distinguish two general strategies which insects evolved to keep energy balance under cold stress conditions. First one, characteristic for freeze tolerant insects is reducing of more oxidative metabolism in favor of less energy-efficient anaerobic respiration. It is accompanied by lowering of COX activity, suppression of fatty acid oxidation (e.g. *E. solidaginis*) and decrease in CS activity but without losing respiratory capacity (e.g. diapausing *C. costata).* In turn, chill susceptible (e.g. *A. diaperinus*) and freeze avoiding insects (e.g. *E. sccuderiana)* defend against stress in similar manner*.* Under cold stress insects relaying on this strategy slowdown mitochondrial processes, including oxygen consumption and ATP production, and use aerobic oxidation of fatty acids as a most useful source of energy.

## Oxidative stress

Mitochondria, respiring at normal aerobic conditions, generate reactive oxygen species (ROS). Insects faced with cold stress change their cellular metabolism, which often results in increased accumulation of ROS by mitochondria. Consequently, the mitochondrial antioxidant system during cold stress will be modulated. The dominant ROS, superoxide anion (O_2_^–·^) is generated mainly from electron leakage from complexes I (exogenous NADH dehydrogenase) and III (ubiquinone, UQ/cytochrome b complex) during electron transport through respiratory chain [118]. O_2_^–·^ can be metabolized to hydrogen peroxide (H_2_O_2_) by superoxide dismutase (Mn-SOD and Cu, Zn-SOD), and then transformed via catalase (CAT) or glutathione peroxidase (GPx). Environmental stressors, including cold, can disturb the balance between ROS production and their detoxification leading to oxidative stress. ROS act as signaling molecules within the cell, but their higher concentrations become damaging to molecules such as DNA, proteins or lipids [10]. The chill-susceptible and -tolerant insects will most likely increase the activity of antioxidant machinery to counteract the detrimental effects of ROS accumulation. Jia et al. [112] showed that in *Apis cerana,* genes encoding syntheses of cytochrome c oxidase (SCOs) are differentially expressed under different environmental cues. In many organisms, SCO proteins are required for COX activity. During severe cold stress (4 °C) *AccSCO*_*2*_ transcription was induced, which indicates that it may play a role in scavenging superoxide anions generated under cold stress conditions to protect *A. cerana* from ROS-induced damages [112]. The lack of or knockdown of this gene leads to a reduction in the activity of antioxidant enzymes, such as SOD which is typically overexpressed during cold stress [119]. Oxidative stress aroused during cold stress can also lead to increased amounts of SOD and CAT in insects transferred to warmer conditions as was shown in *Musca domestica* [120] and *Locusta migratoria* [121], thus, relieving oxidative injury. Lalouette et al. [115] evidenced that cold exposure causes oxidative damage in adult *A. diaperinus* beetles, and that a warm recovery period during fluctuating thermal regimes (FTR) activates the antioxidant mechanism allowing repair of cold-induced damages. Such mechanisms (rewarming pulses) reduce the amount of chill injuries and increase survivability of beetles. Increased severity of cold stress (0–15 °C) during fluctuating thermal regimes caused a decrease in the total glutathione pool (GSHt), whereas SOD levels elevated during the warm recovery period. Similarly, the response of the antioxidant system at the end of the warm period for FTR was sufficient to cope with ROS generation, observed as an increase in GSH:GSSG which is a ratio of reduced to oxidized glutathione illustrating the antioxidant capacity of the cell. [115].

Augmented production of ROS triggers mechanisms leading to reduction of their potentially damaging effect in the cell either by activation of antioxidants [106] or by activation of UCPs. It was evidenced that UCPs crucial role is reducing of free radical generation [122, 123]. In the fat body and muscle mitochondria of *G.* *coquereliana* cockroach, activation of UCP4 lowered the level of superoxide anion [102]. Similarly, Alves-Bezerra et al. [124] indicate that UCP4 may be involved in antioxidant defense in *Rhodnius prolixus* thus protecting cells during oxidative stress aroused during cold exposure. Such mild uncoupling may be a control mechanism of free radical production also, in marine invertebrates [118].

Low temperature or entry into diapause reduces organismal demand on mitochondrial oxygen-based ATP production. In freeze-tolerant species *E. solidagini*s suppressing of mitochondrial respiration during winter months had beneficial functions for reduction of ROS production by respiratory chain and protecting of mitochondrial membranes during freezing by the concentration of intracellular and intramitochondrial fluids [70]. Joanisse and Storey [114, 125] also observed reduced efficiency of antioxidant enzymes in overwintering larvae of this species. Indicating that larvae of *E. solidagini*s do not face increased challenge from oxidative stress during the numerous freeze–thaw cycles they experience during winter months. Also, freeze-tolerant *E. solidaginis* seems to lack or have very minimal activity of xanthine oxidase, an enzyme implicated in ROS generation, as it uses oxygen as an oxidizing agent which leads to superoxide formation as its reduction product. The absence of such a conversion in the freeze-tolerant larvae could indicate an adaptive strategy to reduce ROS during oxygen reperfusion during thawing [114]. In another freeze-tolerant insect, *Belgica antarctica* the freezing causes no significant oxidative damage, while the total antioxidant capacity remains at the constant level when the insects are stressed [126].

On the other hand, increased winter activities of antioxidant enzymes (*inter alia* SOD, CAT and GST) in freeze-avoiding *E. scudderiana* suggest that these larvae must defend against detrimental effects of ROS in similar manner as the chill susceptible insects. Even though the metabolism of *E. scuderiana* is decreased during freeze-stress, it is still aerobic, which seems to be enough for ROS production at damaging level and increased antioxidant activity to prevent these damages [114].

Therefore, the insects subjected to cold stress rely on two strategies to avoid oxidative stress. It is an increase in the activity of antioxidant enzymes, as is the case with chill-susceptible (in the case of these insects also mild uncoupling by UCP) and freeze-avoiding insects, or they slow down their metabolism, thus reducing the production of ROS and protecting their cells, as evidenced for freeze-tolerant ones.

## Heat shock proteins

In addition to the antioxidant stress system, heat shock proteins (HSPs) can also provide a protective function to stressed cells. Although they are named heat shock proteins, they also take part in response to many other biotic and abiotic stresses [127, 128]. HSPs are a part of cell preservation strategy under low temperature and their role in protecting insects against stress, either temperature or desiccation, is studied intensively [106, 129–133]. HSPs are omnipresent in animal cells, some are constitutive and others are inducible in response to environmental stressors such as a cold [134–136]. *HSP* genes encode a family of highly conserved proteins, which act as chaperones to stabilize and refold denatured proteins, preventing the formation of cytotoxic aggregates [134–136], hence, the expression and activity of heat shock proteins will change during cold stress.

The popularity of *HSPs* in molecular studies is large due to the high conservation of these genes at the molecular level and therefore, relative ease to clone and evaluate in different species, even where there is limited or no genome data available for that species [106, 129–131]. Animal forms of HSPs are found ubiquitously and can be divided based on their localization in the cell: e.g., endoplasmic reticulum (ER), mitochondrial, and cytosolic forms [137, 138]. In the mitochondria, HSP70, HSP60, HSP20, and HSP10 proteins have been detected. Some researchers focus their studies on the gene expression profiles of *HSP* genes while others evaluate the abundance of the proteins. The ideal would be combining both approaches. Hence, in this section italicized names refer to genes (*HSP)*, as opposed to plain type names which refer to research conducted on proteins (HSP).

Lu et al. [107] demonstrated that during cold stress mitochondrial-specific *HSP60* from chill-tolerant *Frankliniella occidentalis* is clearly up-regulated in a time-dependent manner. Similarly, frozen larvae of *B. antarctica* tend to have higher expression level of *HSP60* [53] and one-third higher during recovery after rapid cold hardening (RCH) [105]. The slight upregulation of *HSP60* can be also observed in *Monochamus alternatus,* freeze-avoiding beetle [109]. In the *Galeruca daurica* there is also clear up-regulation of *HSP60*, the level of which depends on the temperature [108]. On the other hand, the expression pattern of *HSP60* does not differ under cold/freezing stress or even tends to be down-regulated in *Leptinotarsa decemlineata* [104] as it is in other taxa [139]. Similar results were shown in *D. melanogaster* in which Colinet et al. [106] also did not observe any changes in *HSP60* expression pattern during cold stress and recovery period.

Interestingly, they did observe changes in other mitochondrial *HSPs* genes*, HSP22* and *HSP23,* so called small HSPs (sHSP) [106, 140]. Overexpression of both s*HSPs* was noted during the recovery after 9 h of cold stress [106]. The overexpression of *sHSP22* was also noted in diapausing, cold-acclimated *C. costata* larvae, one of the most cold-hardy animals on Earth [76]. As with *HSP60*, studies on *sHSPs* showed contradictory results. In *S. crassipalpis*, the *HSP23* is overexpressed in non-diapausing individuals in response to cold shock (− 10 °C) [111]. Whereas during recovery from cold stress (3 h at 0 °C) Sinclair et al. [110] did not observe any changes in *D. melanogaster*. Colinet et al. [106] suggested that long exposure to stress is needed to obtain a response from these genes, as they stated “*Perhaps, as for Hsp70, it takes several hours under mild cold stress to obtain a response in sHsp genes.*”. However, they also note that in the same species, Qin et al. [141] reported overexpression of *sHSPs* during 30 min of recovery after only 2 h of cold stress. We believe that the differences in the expression profile of these genes may be caused by the climatic zone of the insect's origin. Colinet et al. [106] conducted their studies on flies from a rather tropical zone (Innisfail, Australian east coast) whereas Sinclair et al. [110] from temperate (Terhune, New Jersey, USA). Hence, authors working in the future on this topic should consider the origins of the insects as well as possible differential expression during cold stress and recovery from it. Nevertheless, mitochondrial *(s)HSPs* are essential for insect survival as the RNAi experiments showed that silencing of *HSP22 and HSP23* reduces insect survival during cold stress [142]. Additionally, HSP22 protein takes part in protecting cells against oxidative injuries [143]. The activation of these molecules plays a significant role in the secretion of signaling molecules or the induction of tissue regeneration [144]. Therefore, the proper activity of these genes can be very important not so much during cold stress, but during cold stress recovery, and future.

Mitochondrial HSP70 plays a central role in mitochondrial biogenesis. Zhang et al. [113] showed that a member of this family increases its abundance during winter months in *E. solidaginis.* Laboratory induced freezing of the insect (larvae shifted acutely from 3 °C down to − 16 °C and frozen for 24 h), and the laboratory cold treatment (3 °C exposed for 24 h) did not cause changes in protein abundance. Only freeze–thaw treatment (similar to environmental conditions) caused substantial increase, which provided enhancement of mitochondrial chaperone activity comparable to that seen for the cytoplasmic and endoplasmic reticulum [113]. Many authors focus their research on HSP70 protein, however they do not discriminate between the cytosolic and mitochondrial fractions of these proteins, most often measuring their entire pool in the cell. The studies on mitochondrially-specific HSP70 during cold stress is limited. Hence, the studies on different fractions are a promising line of research, as the function of HSPs may be different during recovery time (neutralization of damages after cold stress) and during stress (protection against damages during cold stress) as well as due to their cellular localization.

The HSP60 family of proteins that is specific for mitochondrial localization possess a characteristic (GGM)n repeat motif [139, 145]. HSP60 is known to chaperone nascent polypeptides for their transport from the cytoplasm to the mitochondrial matrix in conjunction with HSP10 that also resides in the mitochondria [146]. Moreover, to its classical chaperone function, mitochondrial HSP60 is critically implicated in the replication and transmission of mitochondrial DNA [147]. Compared to the upregulation of HSP in *E. solidaginis*, mitochondrial HSP60 protein is strongly suppressed over the winter months [113]. It is worth mentioning that this pattern parallels lowered activities of many mitochondrial enzymes e.g., citrate synthase, glutamate dehydrogenase, NAD-isocitrate dehydrogenase, malic enzyme, cytochrome c oxidase and enzymes of fatty acid oxidation that are reduced by 50–65% during cold exposure in *E. solidaginis* [35, 47, 79]. HSP60 can also enhance cold hardiness and promote membrane stability. In *Delia antiqua* it contributes to the enhancement of cold hardiness through repression of the depolymerization of actin filaments at low temperatures [103].

As mentioned above HSP10 is a co-chaperone and work in conjunction with HSP60, exerting its biological functions in diverse conditions [148]. HSP60 and HSP10 form a folding cage through their rings and HSP60–HSP10 complex may accelerate polypeptide folding, denatured protein refolding, and misfolded protein correcting [149–151]. Data on the HSP10 activity in insects is scarce. Understanding the gene expression patterns and its biological interactions with HSP60 and HSP70 would bring us closer to understanding the role of this protein/s in mechanism responsible for cold stress tolerance in insects.

The research on mitochondrial fraction of different HSPs forms is limited, however, the picture emerging from the available studies indicates that, whatever the type of strategy insects use to survive the cold/freeze stress, there is an up-regulation of *(s)HSPs/*(s)HSPs.

## Apoptosis

Mitochondria are crucial for cells and organisms surviving not only because of being an energetic center, but also as regulators of programmed cell death in which they take a significant part [152]. Cold stress is a generator of oxidative stress and affects mitochondria disrupting their functioning, thus it might be supposed, that cold stress is associated with induction of apoptosis via mitochondrial pathways. Mitochondria can switch from the adaptive response to cell death due to energy deficiency and/or the overwhelming mitochondrial or cellular damage [37, 38]. Nevertheless, the knowledge about apoptosis induced by cold stress, especially via mitochondrial pathway, in insect is very scanty. There are only a few papers showing connection between cold, mitochondria and apoptosis. In 2007, Yi et al. [58] showed that cold stress increases the number of apoptotic cells in flight muscles of chill susceptible flies, *D. melanogaster*, which were not cold acclimated. On the other hand, these authors showed that RCH protects the cells against apoptosis and observed that in insects, which were acclimated to a low temperature before cold or freezing treatment, the apoptotic rate was significantly lower than in the group without acclimation. The researchers also analyzed the level of different apoptotic factors. They noticed that two caspases (procaspase-9-like and active caspase-3-like) were present in both groups—cold-acclimated and non-acclimated, but they were least abundant in the first group. Moreover, the apoptotic inhibitor Bcl-2 was down-regulated in cold-shocked group compared to the control and RCH groups [58]. The participation of mitochondria in apoptosis might be associated with mitochondria swelling, and role of mitochondria in the balance of cellular calcium level. Above mentioned are suggested by results obtained by Štětina et al. [59] and Bayley et al. [153]. Štětina et al. [59] showed that in freeze-sensitive (non-diapause) phenotype larvae of fly, *C. costata*, the mitochondria undergo swelling in responses to freezing stress what was not observed in freeze-tolerant (diapausing, cold-acclimated) phenotype. Interestingly, the freezing-induced mitochondrial swelling was abolished by feeding freeze-sensitive larvae on a proline-augmented diet, thus, the authors suggest the stabilizing role of proline against mitochondria swelling. Regrettably, they did not analyze the occurrence of apoptosis in studied insects [59]. While Bayley et al. [153] showed that cold induces an excessive Ca^2+^ influx, increasing the intracellular calcium level in muscle cells of the tropical (chill-susceptible) migratory locust *L. migratoria*. That may induce Ca^2+^-mediated necrosis/apoptosis probably also including mitochondria swelling. Kumarswamy et al. [154] showed that mitochondrial Ca^2+^ overload occurs in apoptotic insect Sf9 cells treated with actinomycin-D. Moreover, the research conducted in *D. melanogaster* has shown that regulation of HSP60, a mitochondrial chaperon responsible for maintaining homeostasis of mitochondrial proteins [155] is essential for caspase-dependent apoptosis [156], while the sHSPs prevent this pathway [157]. Tight regulation of apoptosis is essential for survival as the stimulated activity of caspases is not solely the indicator of apoptosis but besides, it demonstrates nonapoptotic functions i.e., control of a cell shape, cell migration or proliferation.

The data concerning apoptosis induced by cold stress as well as the role of mitochondria in it are very poor. Nevertheless, they support guess that mitochondria play an important role in protection against cold stress as participants and also executors of programed cell death.

## Morphology

In consequence to the abovementioned phenomena, it seems obvious that mitochondrial morphology must undergo significant alterations. Mitochondria are dynamic organelles composed of double membranes [158, 159]. The shape, size and/or the number of mitochondria is extremely plastic and can be modulated by physiological conditions as well as environmental cues [160–162]. In plants and animals such as cold-bodied fish, it is well established that low temperature affects the shape and the number of these organelles and their velocity within the cell [163–165]. However, the number of studies on the effect of low and freezing temperature on morphology and number of insect mitochondria is scarce and to our knowledge, only a handful of researchers have touched this topic. Available data suggest that in insects (freeze-tolerant) the number of mitochondria is reduced [35, 84, 166]. For *G. groenlandica*, drop of temperature down to 0 °C is a signal for starting the dormancy period. They break down mitochondria in response to cold-acclimation (i.e., their number decreases, and they degenerate), and enter diapause as a strategy to conserve energy when the temperature drops down [84, 167].

Another typical symptom in response to stressors is mitochondrial swelling which in consequence leads to dilution of the matrix and finally to rupture of the outer membrane [168, 169] (Fig. 1). Lee et al. [170] showed that in Malpighian tubules of freeze-tolerant *E. solidaginis* larvae subjected to lethal for them temperature of − 55 °C, mitochondria had been swollen and round-shaped in contrast to individuals subjected to sub-lethal freezing at − 22 °C, whose mitochondria had remained rod-shaped. In the same species, *E. solidaginis* and *G. groenlandica*, Levin et al. [166] observed a reduction in the mitochondrial activity in larvae collected in colder seasons (i.e. cold-acclimated individuals), in comparison with the insects collected in summer, which can be the effect of the drop in the number of mitochondria expressed by a decrease in the amount of mtDNA. Štětina et al. [59] tested the effects of freezing stress on mitochondria of *C. costata* larvae. Although in acclimated (diapausing) insects the freezing stress had no effect on the number of mitochondria—contrary to the non-acclimated (non-diapausing) individuals—after acclimation mitochondria were not evenly distributed within the cell, and two sub-populations were distinguished—one located around the nucleus and the other close to the cell periphery. In the same study, the authors observed (although, statistically not significant) “certain tendency” to swelling of mitochondria. Exposing non-acclimated individuals of *C. costata* to freeze stress resulted in ca. 65% mortality, caused enlargement and swelling of mitochondria with dilution of electron-dense material in the matrix [59]. The authors presented electronograms of mitochondria obtained from warm-acclimated and cold-acclimated *C. costata* larvae. The cold-acclimated larvae possessed mitochondria with clearly visible, more numerous cristae. That suggests more intensive oxidative metabolism. The enlarged mitochondria, the loss of matrix density and altered cristae may result from oxidative stress, which in turn can be the effect of low temperature.

The results of our preliminary electron microscopic research on cold-stressed *G. coquereliana*, and applied stereological observations (unpublished) suggest that low temperatures (4 °C) may cause fine ultrastructural changes of the fat body, including mitochondria. Although we did not note significant alterations of the shape or electron density of mitochondria, there was a slight growth of the ratio of the surface of the inner membrane to the outer one, as shown in stereological calculations. This indicates the increased surface of the inner membranes within mitochondria, and, in consequence, intensification of the energy metabolism within mitochondria of the fat body of the cold-exposed insects. Next, the number of glycogen granules within the cytoplasm slightly decreased in the fat body of stressed organisms. This is consistent with our earlier results where short cold stress (3 h, 4 °C), as well as repeated exposure to low temperature (3 × 8 h, 4 °C), lowered the glycogen content in fat body tissue [89, 116]. Fat body plays a crucial role in controlling of storage and utilization of energy reserves in insects. The primary source of energy are lipids [171]. Therefore, altered level of glycogen may appear when the stress is significant. Perhaps, the time of exposure to stress plays a crucial role in the process. Glycogen, in turn, plays important role in the synthesis of sugar alcohols, trehalose and glucose which are mobilized during adaptation to cold [69, 172, 173]. At the same time, glycogen level decreases [174]. Next, glycogen and glucose are crucial substrates for synthesis of glycerol, which is one of the important cryoprotectants [74]. Hence, one may conclude that energetic metabolism plays an important role in response to low temperatures, in insects, and in consequence, significantly affects mitochondrial structure.

## Future directions

Mitochondria play a key role in energy supplying and conversion in all living organisms, including insects. Their activity as well as other cellular organelles and biochemical processes depends on many environmental factors, among them temperature is one of the most important. We know that cold stress may trigger mechanisms leading to decline of mitochondrial oxidative capacity, loss of metabolic homeostasis, depletion of energy substrates, oxidative damage and in consequence cellular injury. On the other hand, mitochondria are capable to counteract and adjust to the effects of cold stress to maintain energetic homeostasis and this response may be varied at different cellular levels (gene expression, protein level, activity of enzymes) and depends on climatic zone or adaptation of insects to cold stress.

Some questions arise, how collapsing of mitochondrial function, especially their capacity for ATP production evoked by low temperature stress is related to thermal limits of insects at cellular and organismal level. How is the correlation between these processes and growth and development of insects under the cold stress and fluctuating temperature conditions? At the time of climate changing, maintaining of energy homeostasis may be crucial for survival of insect species and their distribution at different latitudes. Considering that electron transport system (ETS) is responsible for 80–90% oxidative phosphorylation capacity, it seems crucial to explore, how mitochondria provide energetic requirements in the cells, and this way enable survival of insects during cold stress conditions.

Another interesting venue for the future research is analyzing cellular mechanisms which function depends on the access of ATP such as Na^+^/K^+^-ATPase, protein pump in cellular membrane powered by ATP which allows the cell to maintain mineral–water homeostasis. Poorly understood is the role of insect mitochondria in controlling of calcium homeostasis in the cell and their involvement in apoptotic processes caused by cold. It seems interesting also to elucidate the potential thermogenic role of UCP in insects exposed to low temperature stress, and the engagement of these proteins and other mechanisms in redox homeostasis, which is an important part of stress tolerance in insects. It would be also interesting to make relationship between mitochondrial response and cellular transduction pathways in cold-tolerant and cold-sensitive insects from different geographical zones, by using common methods and experimental conditions. Data obtained so far are ambiguous and do not allow us to draw the final conclusions giving researchers the big task of resolving these issues in the future.

## Data Availability

Not applicable.

## Abbreviations

TCA: Tricarboxylic acid cycle
FTR: Fluctuating thermal regime
CLT: Constant low temperature
PDC: Pyruvate dehydrogenase complex
SCP: Supercooling point
CS: Citrate synthase
MDH: Malate dehydrogenase
LDH: Lactate dehydrogenase
COX: Cytochrome c oxidase
mtDNA: Mitochondrial DNA
HSP: Heat shock protein
RCR: Respiratory control ratio
OCR: Oxygen consumption ratio
CO: Cold-susceptible
CA: Cold-acclimated
IMM: Inner mitochondrial membrane
UCP: Uncoupling protein
ADP/O: Mitochondrial phosphorylation efficiency
FFA: Free fatty acids
PN: Purine nucleotides
ROS: Reactive oxygen species
CAT: Catalase
SOD: Superoxide dismutase
GP: Glutathione peroxidase
SCO: Synthase of cytochrome c oxidase
RCH: Rapid cold hardening
ETS: Electron transport system
